# A multi-institutional retrospective pooled outcome analysis of molecularly annotated pediatric supratentorial *ZFTA-*fused ependymoma

**DOI:** 10.1093/noajnl/vdad057

**Published:** 2023-05-12

**Authors:** Chia Huan Ng, Denise Obrecht, Olivia Wells, Michal Zapotocky, David Sumerauer, Hallie Coltin, Dong-Anh Khuong-Quang, David D Eisenstat, Kathryn M Kinross, Christine L White, Elizabeth M Algar, Amanda Luck, Hendrik Witt, Ulrich Schüller, Martin Mynarek, Torsten Pietsch, Nicolas U Gerber, Martin Benesch, Monika Warmuth-Metz, Rolf Kortmann, Brigitte Bison, Michael D Taylor, Stefan Rutkowski, Stefan M Pfister, David TW Jones, Nicholas G Gottardo, Katja von Hoff, Kristian W Pajtler, Vijay Ramaswamy, Jordan R Hansford

**Affiliations:** Children’s Cancer Centre, Royal Children’s Hospital, Murdoch Children’s Research Institute, University of Melbourne, Melbourne, Australia; University Medical Center Hamburg-Eppendorf, Hamburg, Germany; Children’s Cancer Centre, Royal Children’s Hospital, Murdoch Children’s Research Institute, University of Melbourne, Melbourne, Australia; Department of Paediatric Haematology and Oncology, Charles University, 2nd Faculty of Medicine and Faculty Hospital Motol, Prague; University Medical Center Hamburg-Eppendorf, Hamburg, Germany; Department of Paediatric Haematology and Oncology, Charles University, 2nd Faculty of Medicine and Faculty Hospital Motol, Prague; Developmental and Stem Cell Biology, Labatt Brain Tumour Research Centre, The Hospital for Sick Children, Toronto, Canada; Division of Haematology/Oncology, Hospital for Sick Children, Toronto, ON, Canada; Division of Pediatric Hematology-Oncology, Charles-Bruneau Cancer Centre, CHU Sainte-Justine, University of Montreal, Montreal, Quebec, Canada; Children’s Cancer Centre, Royal Children’s Hospital, Murdoch Children’s Research Institute, University of Melbourne, Melbourne, Australia; Children’s Cancer Centre, Royal Children’s Hospital, Murdoch Children’s Research Institute, University of Melbourne, Melbourne, Australia; Hudson Institute of Medical Research, Melbourne, Australia; Department of Molecular and Translational Science, Monash University, Melbourne, Australia; Hudson Institute of Medical Research, Melbourne, Australia; Australia and New Zealand Children’s Haematology/Oncology Group, Melbourne, Australia; Hudson Institute of Medical Research, Melbourne, Australia; Department of Molecular and Translational Science, Monash University, Melbourne, Australia; Victorian Clinical Genetics Services, Melbourne, Australia; Hudson Institute of Medical Research, Melbourne, Australia; Department of Molecular and Translational Science, Monash University, Melbourne, Australia; Michael Rice Cancer Centre, Women’s and Children’s Hospital; South Australian Health and Medical Research Institute, Adelaide, Australia; German Cancer Research Centre, DKFZ, Heidelberg, Germany; University Medical Center Hamburg-Eppendorf, Hamburg, Germany; University Medical Center Hamburg-Eppendorf, Hamburg, Germany; Department of Neuropathology and DGNN Brain Tumor Reference Center, University Bonn Medical Centre, Germany; Children’s Hospital of Zurich, Switzerland; Medical University of Graz, Austria; University Hospital Leipzig, Leipzig, Germany; University Hospital Leipzig, Leipzig, Germany; University of Wuerzburg, Würzburg; Developmental and Stem Cell Biology, Labatt Brain Tumour Research Centre, The Hospital for Sick Children, Toronto, Canada; University Medical Center Hamburg-Eppendorf, Hamburg, Germany; Hopp Children’s Cancer Center Heidelberg (KiTZ) and Division of Pediatric Neurooncology, German Cancer Research Center (DKFZ), Heidelberg, Germany; Department of Pediatric Oncology, Hematology, and Immunology, University Hospital Heidelberg, Heidelberg, Germany; Hopp Children’s Cancer Center Heidelberg (KiTZ), Pediatric Glioma Research Group, German Cancer Research Center (DKFZ), Heidelberg, Germany; Hudson Institute of Medical Research, Melbourne, Australia; Perth Children’s Hospital, Telethon Kid’s Institute, Western Australia, Perth, Australia; Charité Universitätsmedizin Berlin, Germany; Hopp Children’s Cancer Center Heidelberg (KiTZ) and Division of Pediatric Neurooncology, German Cancer Research Center (DKFZ), Heidelberg, Germany; Developmental and Stem Cell Biology, Labatt Brain Tumour Research Centre, The Hospital for Sick Children, Toronto, Canada; Division of Haematology/Oncology, Hospital for Sick Children, Toronto, ON, Canada; Departments of Medical Biophysics and Pediatrics, University of Toronto, Toronto, ON, Canada; Children’s Cancer Centre, Royal Children’s Hospital, Murdoch Children’s Research Institute, University of Melbourne, Melbourne, Australia; Hudson Institute of Medical Research, Melbourne, Australia; Michael Rice Cancer Centre, Women’s and Children’s Hospital; South Australia Health and Medical Research Institute; South Australia ImmunoGENomics Cancer Institute, University of Adelaide, Adelaide SA, Australia

**Keywords:** central nervous system tumors, molecular, pediatric, supratentorial ependymoma, ZFTA-RELA

## Abstract

**Background:**

*ZFTA-RELA* (formerly known as *c11orf-RELA)* fused supratentorial ependymoma (*ZFTA*fus ST-EPN) has been recognized as a novel entity in the 2016 WHO classification of CNS tumors and further defined in the recent 2021 edition. *ZFTA*fus ST-EPN was reported to portend poorer prognosis when compared to its counterpart, *YAP1* ST-EPN in some previously published series. The aim of this study was to determine the treatment outcome of molecularly confirmed and conventionally treated *ZFTA*fus ST-EPN patients treated in multiple institutions.

**Methods:**

We conducted a retrospective analysis of all pediatric patients with molecularly confirmed *ZFTA*fus ST-EPN patients treated in multiple institutions in 5 different countries (Australia, Canada, Germany, Switzerland, and Czechia). Survival outcomes were analyzed and correlated with clinical characteristics and treatment approaches.

**Results:**

A total of 108 patients were collated from multiple institutions in 5 different countries across three continents. We found across the entire cohort that the 5- and 10-year PFS were 65% and 63%, respectively. The 5- and 10-year OS of this cohort of patients were 87% and 73%. The rates of gross total resection (GTR) were high with 84 out of 108 (77.8%) patients achieving GTR. The vast majority of patients also received post-operative radiotherapy, 98 out of 108 (90.7%). Chemotherapy did not appear to provide any survival benefit in our patient cohort.

**Conclusion:**

This is the largest study to date of contemporaneously treated molecularly confirmed *ZFTA*fus ST-EPN patients which identified markedly improved survival outcomes compared to previously published series. This study also re-emphasizes the importance of maximal surgical resection in achieving optimal outcomes in pediatric patients with supratentorial ependymoma.

**Key Points**

1. This retrospective analysis of a large cohort of *ZFTA*fus ST-EPN identified markedly improved survival outcomes compared to some previously published series.
2. Maximal safe surgical resection remains to be an important outcome predictor in the treatment of pediatric *ZFTA*fus ST-EPN.
3. Chemotherapy did not show survival benefit in our cohort of patients.

**Importance of the Study**

This study reported more favorable outcome in a large cohort of contemporaneously treated patients with *ZFTA*fus ST-EPN compared to previously published series. It provides updates to our knowledge of this tumor’s behavior. Albeit a retrospective study, it re-emphasizes the importance of maximal surgical resection and confirms the relative lack of benefit of chemotherapy in this tumor. This study will aid clinicians in counseling and decision making for this class of tumor and make suggestions that clinical trialists can consider for the next generation of ependymoma studies.

With the recent advancement of (epi)genomic profiling technology in pediatric oncology, molecular classifications have supplanted conventional histopathological or clinical classification in many tumor types.^1^ Pediatric ependymoma is one of the central nervous system (CNS) tumors whereby development of in-depth understanding of its driver of tumorigenesis from a genomic standpoint has provided significant insight into its prognosis in the recent decade.

Ependymomas are neuroepithelial tumors that can arise in all compartments of the CNS at all ages but are most common in childhood, especially in young children. The majority (>90%) of pediatric ependymomas occur intracranially either in the supratentorial (ST) compartment or posterior fossa (PF). In their seminal paper, Pajtler et al. identified 9 molecular subgroups in a large cohort of 500 ependymal tumors.^2^ Within the supratentorial compartment, ependymomas can be driven by distinct gene fusions initially described as involving the NF-kB subunit *RELA, c11orf-* or the HIPPO signaling regulator *YAP1*.^3^ Since these initial descriptions, it was found that the open reading frame component of the *c11orf95-RELA* fusion is the recurrent component of most variants of supratentorial disease. *ZFTA-RELA* fused supratentorial ependymoma (*ZFTA*fus ST-EPN) characterized by an oncogenic fusion between zinc finger translocation associated (*ZFTA*, formerly known as *C11orf95*) and in most cases v-rel avian reticuloendotheliosis viral oncogene homolog A (*RELA*).^3–6^ Other alternative genes fused to *ZFTA* have been described in additional cases.^5,7^*ZFTA*fus ST-EPN, account for more than 70% of supratentorial ependymomas and primarily occur in children and young adults. A retrospective report found that *ZFTA*fus ST-EPN was associated with a poor 10-year overall survival (OS) of only 49% and progression free survival (PFS) of 19%.^2^ Previous reports on pre-clinical mouse models have shown that *C11orf95-RELA* fusion is potent oncogenes that most may transform neural stem cells by driving an aberrant NF-kB transcription program. Pathological nuclear accumulation of *p65-RELA* subsequently occurs which represents the hallmark of ST-RELA-EPN tumors.^8,9^ The 3 complementary reports by Kupp et al. in 2021 provided important and novel insight into the molecular and cancer phenotype characteristics of *ZFTA*fus tumors.^4–6^ The management of ependymoma is evolving. The current mainstays of treatment for pediatric ependymal tumors include maximal safe surgical resection followed by conformal radiotherapy.^10–12^ The role of chemotherapy remains contentious. To date, no chemotherapeutic regimen has proven to have any survival benefit for these patients and is currently under investigation by both the COG and International Society of Pediatric Oncology (SIOP) (NCT02265770).

Given the increased appreciation of this relatively new tumor class, we sought to better understand and define the clinical behavior of contemporaneously treated and molecularly confirmed *ZFTA*fus tumors to help guide future therapeutic interventions. We retrospectively collected and collated the molecular and clinical features of 108 patients treated between 1995 and 2020 at institutions across 3 continents (Europe, North America, and Australia). Herein, we present our study showing markedly improved outcomes than those reported in the seminal ependymoma paper for children with *ZFTA*fus - ST EPN.^2^ This study will aid clinicians in counseling and decision-making for this class of tumor and make suggestions that clinical trialists can consider for the next generation of ependymoma studies.

## Materials and Methods

We conducted a retrospective analysis of all pediatric patients with molecularly confirmed and reported supratentorial ependymomas treated in multiple institutions in 5 countries (Australia, Canada, Germany, Switzerland, and Czechia). All patients with molecularly classified *ZFTAfus* ST-EPN diagnosed between 1995 and 2020 were included for analysis. Non-ZFTA-classified ependymal fusions were excluded from this analysis, including YAP fusions. In our local molecular characterization program, no patients with YAP fusions were found in over 5 years (unpublished) and in the international molecular profiling series, only one case was found.^5^ As such, our capacity to collect substantive and informative data on these entities would be meaningless. Demographic information, extent of surgical resection, histological grading according to World Health Organization (WHO) classification of CNS tumors, use of radiotherapy and/or chemotherapy, disease recurrence, treatment at recurrence, and clinical outcome data were collected. Patients were identified through local review of pathology databases, electronic, or paper medical records unique to each institution. The data was collected at each collaborating site through patient chart review at the respective institutions. Data from each institution was then collated for final analysis. This study was approved by local and collaborating institutions’ research ethics boards.

PFS and OS were analyzed by the Kaplan–Meier method and *P*-values were reported using the log-rank test. Associations between covariates and risk groups were tested by the Fishers exact test. Univariable and multivariable Cox proportional hazard regression was used to estimate hazard ratios including 95% confidence intervals. All statistical analyses were performed in the R statistical environment (v4.2.1), using R packages survival (v3.4-0), and ggplot2 (v3.3.6).

## Results

Using DNA methylation profiling, both retrospectively and prospectively, we identified a total of 108 pediatric patients with ZFTAfus supratentorial ependymoma Patients were diagnosed and treated between 1995 and 2020 at the author’s respective institutions. The patient’s clinical characteristics are summarized in Table 1.

**Table 1.** Clinical Characteristics of *ZFTA-RELA* Fused ST-EPN Patients

Characteristic	Patients (*N* = 108)
Sex	
Male – no. (%)	65 (60.2%)
Female – no. (%)	43 (39.8%)
Age	
Median, y	6 y 7 months
Range, y	5 months–18 y 7 months
WHO grading	
Grade II	14 (13%)
Grade III	73 (67.6%)
unknown	21 (19.4%)
Extent of surgical resection	
Gross total resection (GTR)	84 (77.8%)
Subtotal resection (STR)	24 (22.2%)
Radiotherapy	
Yes	98 (90.7%)
No	10 (9.3%)
Chemotherapy	
Yes	63 (58.3%)
E-HIT Stratum A	29 (26.9%)
E-HIT Stratum B	16 (14.8%)
E-HIT Stratum C	4 (3.7%)
ACNS 0831	5 (4.6%)
ICE	3 (2.8%)
Personalized treatment	2 (1.9%)
HIT 91	1 (0.9%)
CCLG 2007	1 (0.9%)
SIOP ependymoma II	1 (0.9%)
No	45 (41.7%)
Relapse/recurrence disease	
Local	21 (19.4%)
Distant	3 (2.8%)
Combined local/distant	1 (0.9%)
Unknown	14 (13%)

There was a male predominance with 65 (60.2%) male patients and 43 (39.8%) female patients. Median age at diagnosis was 6 years 7 months (range: 5 months–18 years 7 months). The majority of the tumors were classed as histopathologic WHO grade III (14 grade II (13%); 73 grade III (67.6%) and 21 unknown (19.4%)). 84 (77.8%) patients underwent gross total resection (GTR) and 24 (22.2%) patients underwent subtotal resection (STR). The vast majority of patients, 90.7% (98/108), received radiotherapy post-operatively with 10 (9.3%) patients not. A total of 63 (58.3%) patients received multiagent chemotherapy, whilst 45 (41.7%) patients did not receive any chemotherapy. The chemotherapy regimens varied according to the institutions that the patients were treated in. Forty nine (45.7%) patients were treated on E-HIT series in 3 different stratums (Stratum A *n* = 29; Stratum B *n* = 16; Stratum C *n* = 4). Five (4.6%) patients were treated as per ACNS 0831. Three (2.8%) patients received ICE (Ifosfamide, Carboplatin, and Etoposide) chemotherapy regimen. Two (1.9%) patients were treated on personalized treatment as per their respective treating physicians. There were individual patients who were treated on HIT91, CCLG 2007, and SIOP ependymoma II respectively.

With a median follow-up time of 5.69 years (range: 0.23 years–20.46 years), a total of 25 patients (23%) relapsed. Fourteen patients did not have sufficient follow-up data to ascertain their relapse status. The majority of relapses 21/25 (84%) were local, with 3 (12%) patients with distant relapses and 1 (4%) patient with combined local and distant relapses.

**Figure 1.**
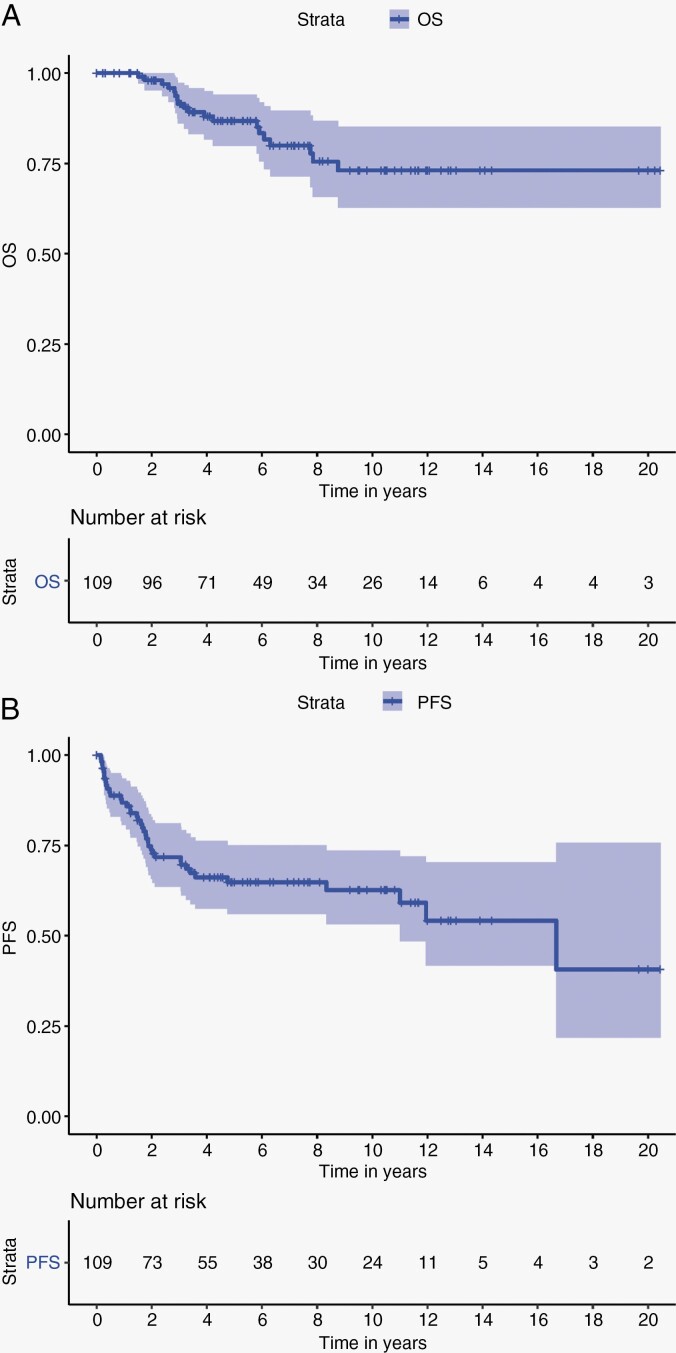
(A) Overall survival of whole cohort. (B) Progression free survival of whole cohort.

**Figure 2.**
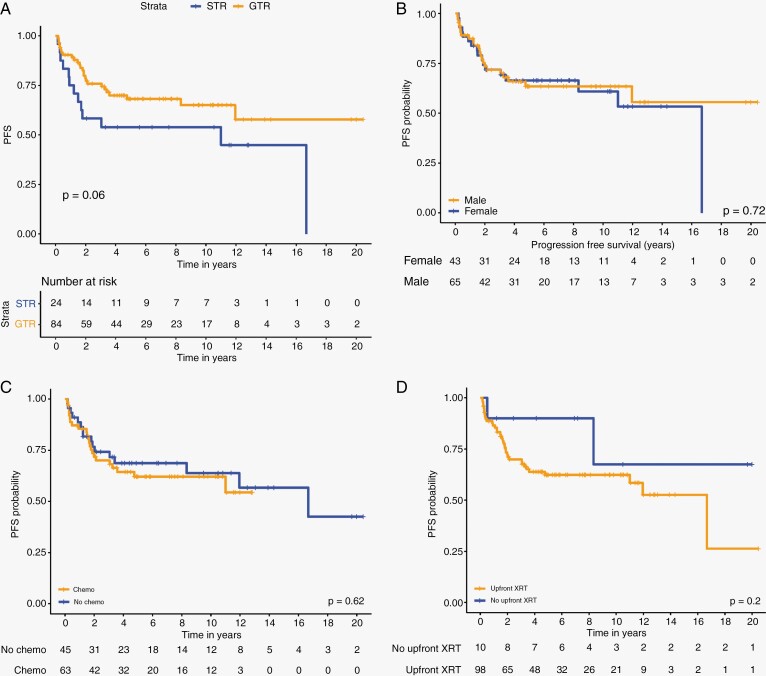
(A) PFS analysis for patients undergoing GTR vs. STR. (B) PFS analysis by gender. (C) PFS analysis for patients by use of chemotherapy. (D) PFS analysis by use of radiation therapy.

The OS at 1, 3, 5, and 10 years for the whole cohort are 100%, 91%, 87%, and 73% respectively (Figure 1a). The PFS at 1, 3, 5, and 10 years of the whole cohort are 87%, 72%, 65%, and 63% respectively (Figure 1b). Univariable analysis revealed that patients who underwent a GTR had a 5-year PFS of 68.1% (95% CI; 0.582–0.797) compared with 53% (95% CI; 0.371–0.782) for patients who underwent STR (*P* = .06) (Figure 2A). Age at diagnosis, gender (Figure 2B), upfront chemotherapy (Fig 2C), receipt of upfront radiotherapy (Figure 2D), and WHO status were not predictors of inferior outcome (Table 2). Multivariable analysis revealed a strong trend to poor survival with a STR compared to those with a GTR (HR 1.88; 95% CI 0.9633–3.66, *P* = .06) (Table 3).

**Table 2.** Univariable Analysis of Survival in ST-RELA

Variable	HR	95% CI	*P*-value
Progression **f**ree **s**urvival (n=108)			
Age	1.03	0.96–1.09	.44
Incomplete **r**esection	1.88	0.96–3.66	.06
Upfront **r**adiotherapy	2.50	0.59–10.61	.21
Male **g**ender	0.89	0.47–1.68	.72
Chemotherapy	1.18	0.62–2.27	.62
WHO Grade III	1.65	0.56–4.82	.36

**Table 3.** Multivariable Analysis of Survival in ST-RELA

Variable	HR	95% CI	*P*-value
*Progression* ***f*** *ree* ***s*** *urvival (n = 108)*			
Age	1.02	0.96–1.10	.47
Incomplete **r**esection	1.22	0.55–2.71	.62
Upfront **r**adiotherapy	2.30	0.44–12.12	.32
Male **g**ender	0.75	0.37–1.52	.43
Chemotherapy	0.96	0.44–2.07	.92
WHO Grade III	1.10	0.31–3.91	.88

Multivariable analysis performed on the entire cohort of patients did not find any difference in survival according to age, sex, or receipt of post-operative radiotherapy. A separate multivariable analysis was also performed on patients who underwent GTR to investigate the impact of chemotherapy on survival outcomes. For this group of patients, no statistically significant difference was seen between patients who received chemotherapy vs. those who did not (HR: 1.15; 95% CI (0.52–2.56), *P* = .73) (Table 3).

## Discussion

There has been an explosion and rapid increase in knowledge over the past decade in the understanding of the molecular features of CNS tumors and their genetic drivers. Due to the high rates of interobserver variability and its lack of predictability in prognosticating patients’ outcomes in some studies, traditional histopathological grading has been slowly supplemented and in some tumor types supplanted by molecular subgrouping in risk stratifying patients for their management.^2^*ZFTA*fus ST-EPN was identified as a novel entity in the 2016 WHO classification of CNS tumors.^13^ Since then, there has been an increasing understanding of its clinical behavior and treatment outcome. In Pajtler’s published large series on the molecular classification of 500 ependymal tumors, 88 out of 500 patients had *ZFTA*fus tumors.^2^ Collectively, these patients had a much inferior outcome when compared to the other ependymoma subgroups with a 10-year PFS of 19% and 10-year OS of 49%. This retrospective series was collected from multiple institutions over decades with little clinical data. As a seminal publication on ependymoma, it has since been believed that *ZFTA*fus ST-EPN portends poor prognosis in contrast to historical studies with markedly better outcomes.^11,12,14,15^ Hukin and Palma reported on 8 and 6 patients with supratentorial ependymoma treated with surgery only.^14,15^ They showed that 12/14 were free of disease without intervention at the time of their publications.^14,15^ Merchant *et al.* reported on the St Jude experience using conformal radiation therapy +/- chemotherapy in upfront ependymoma treatment.^11^ In this prospective study, 31 supratentorial patients were enrolled with 5-year EFS of 82.9% (CI: 66.6–99.2), 5-year OS of 89.5% (CI: 76.8–100.0), and a hazard ratio of 0.52 (CI: 0.20–1.32). More recently, the Children’s Oncology Group (COG) reported on the ACNS0121 study including *ZFTA*fus ST-EPN. This study included enrollment of a cohort of ST-EPN with GTR and classical histology that had expectant observation only postoperatively.^12^ Eleven patients were enrolled and at the time of their publication, the 5-year EFS was 61.4% (95% CI, 33.2% to 89.6%). Local control was achieved in 6 patients (54.55%); local failure occurred in 4 patients (36.36%), and local and distant failure occurred in 1 patient (9.09%). Importantly the 5-year OS was 100%. Following these studies, Upadayayay and colleagues reported on infants and young children treated at St Jude in the SJYC07 infant study.^16^ They showed a PFS of 83.1% (+/- 17%) with only 1 of 8 patients dying from disease. In line with these findings, Jünger et al. reported a 5-year OS of 92.6% and a 5-year PFS of 74.1% in a HIT ependymoma cohort of 54 patients with *ZFTA-RELA* fusion-positive ependymoma.^9^ Given these findings, it is possible that many patients with *ZFTA*fus ST-EPN are being over-treated. Similar to the COG ACNS0121 study, prospective de-escalation of therapy for this tumor class might be considered.

We have also observed that patients who achieved GTR for their primary tumor have higher PFS as compared to their counterparts who only achieved STR, 5-year PFS 68.1% vs. 53% (*P* = .06), although this did not reach statistical significance. This observation is consistent with previously published cohorts again emphasizing the importance of achieving maximal surgical resection safely for patients with ST-EPN.

The implementation of systematic postoperative radiotherapy in clinical trials during the past 20 years has increased the proportion of patients attaining durable disease control with excellent results.^17^ 90.7% of the patients in our cohort received radiotherapy again accentuating its contribution to the favorable overall outcome as compared to Pajtler’s paper whereby 74% of ZFTAfus EPN received postoperative radiotherapy. In combination, a higher rate of GTR followed by higher rate of radiotherapy provided superior survival outcomes in this current cohort of patients. This is an excellent reflection of the result of advances in surgery and radiotherapy through new technologies, increased participation in clinical trials, more centers with pediatric neuro-oncology expertise, improved care, and better collaboration among investigators.^17^ Interestingly, in our cohort, patients not receiving radiation therapy had similar PFS to those that received radiation therapy (Figure 2B). Though the numbers are small and the retrospective nature of our cohort introduces bias, this may suggest a subgroup of patients in whom surgery only approaches might be considered prospectively. Consistent with this finding, Merchant *et al* showed excellent outcomes for a small subset of prospectively enrolled ST-EPN on ACNS0121.^12^ Given the known neurocognitive issues young children in particular acquire with time post radiation therapy, this will be an important question to answer prospectively in the next series of ependymoma studies.

As for the utility of chemotherapy in the management of pediatric ependymoma, we did not find any differences in survival outcomes for those patients who received multi-agent chemotherapy. With 58.3% of the cohort had received chemotherapy with no improvement in survival outcomes (hazard ratio of 0.98), it is very possible we are overtreating children with this entity. This observation is consistent with previously published studies in ependymoma. Traditionally, investigators have explored the utility of chemotherapy in very young patients to avoid or delay irradiation. Through several international studies, its efficacy remains indeterminate.^18–21^ The neuro-oncology community anxiously awaits the publication of large collaborative group studies on ependymoma from the COG and SIOP-Europe conducted in the past decade.

With molecular biomarkers gaining importance in providing ancillary and diagnostic information, WHO CNS tumor classification 2021 has incorporated numerous molecular changes with clinicopathologic utility that are important for the most accurate classification.^22^ Ependymomas should be classified by anatomic site and by molecular group or an associated genetic alteration so that classification of the disease reflects its underlying biology. As such ST-EPN can be classified according to their key diagnostic gene i.e., *ZFTA, RELA, YAP1, MAML2*. Resonating cIMPACT-NOW update 7’s recommendation, an integrated and tiered approach to reporting the diagnosis is advocated for capturing information on molecular characteristics alongside histopathological features.^23^ Molecular subclassification is expected to significantly support treatment decisions and simplify risk stratification processes in the immediate future and should impact clinical trial design and operation in both children and adults.^24^ Despite the increasing importance of molecular characterization on ependymal tumors, DNA methylation studies or gene panel sequencing are not always readily available for diagnostic neuropathologists worldwide. Thankfully, *ZFTA*fus tumors can be diagnosed with specific FISH break apart probes making diagnosis much easier and robust. The majority of ZFTAfus tumors carry fusions with *RELA* and these tumors show strong nuclear accumulation of p65-RelA protein detectable by immunohistochemistry. Both methods, immunohistochemistry as well as FISH can be performed in most diagnostic units worldwide.^25^ It is important to emphasize, while FISH is excellent for *ZFTAfus* tumors, they do not reliably diagnose other known fusions.

Though our study adds to the knowledge about this tumor’s behavior, it does have limitations. Retrospective studies by nature lack knowledge of the treatment intent and decision-making around the timing of surgery, chemo- and radiotherapy. As well, the treatment approaches were heterogeneous across contributing centers making conclusions about the use of chemotherapy difficult. Since commencement of this study, other risk factors for this group and other ependymomas have emerged including *CDK2NA*^9^ and 1q/6q status^26^ that were not examined and will need confirmation. Prospective, worldwide clinical trials are critical as we further define treatment risk and disease stratification to standardize and harmonize therapy for children with *ZFTA*fus tumors.

Moving forward, we have entered an era whereby molecular genetic information is inseparable from histological and clinical information in treating patients with various tumors. We have reported the largest cohort to date of contemporaneously treated patients *ZFTA*fus ST-EPN and demonstrated more favorable survival outcomes compared to previously published series. High rates of GTR in particular likely have contributed to the patients’ outcomes. Given these findings, our team strongly advocates for second look surgery in cases of less than GTR where safe. Chemotherapy did not appear to provide any survival benefit in this cohort of patients. The role of radiotherapy remains unclear as the small number of patients in our series that did not receive radiation therapy did exceptionally well. International prospective clinical trial incorporating molecular risk stratification is required to further evaluate these findings, perhaps with the inclusion of new and novel agents as we learn more about the biology of this new entity.
